# Supplementary material for: Colon length in pediatric health and constipation measured using magnetic resonance imaging and three dimensional skeletonization
Source: PLoS One. 2024 Jan 2;19(1):e0296311. doi: 10.1371/journal.pone.0296311 (PMC10760671; doi:10.1371/journal.pone.0296311)
Supplement: S1 Table — Age, sex, height, weight and body mass index (BMI) for the participants in the functional constipation patient group and the healthy control group. (PDF) [file pone.0296311.s001.pdf]

Supplement 1 Table: Demographic characteristics of the participants. Age, sex, height, weight and body mass index (BMI) for the participants in the functional constipation patient group and the healthy control group.

ID	Group	Age	Sex	Height (m)	Weight (Kg)	BMI (kg/m^2)
1	Patient	8	Male	1.30	60	35.5
2	Patient	11	Male	1.67	99	35.5
3	Patient	10	Female	1.05	41	37.1
4	Patient	13	Female	1.64	60	22.3
5	Patient	7	Male	1.28	25	15.5
6	Patient	9	Female	1.30	48	21.4
7	Patient	10	Male	1.20	34	23.6
8	Patient	16	Female	1.55	68	28.3
9	Patient	10	Female	1.28	22	13.2
10	Patient	11	Male	1.40	34	17.3
11	Patient	11	Female	1.20	35	24.3
12	Patient	18	Female	1.25	52	33.3
13	Patient	13	Male	1.54	60	25.3
14	Patient	13	Male	1.54	42	17.7
15	Patient	14	Female	1.64	63	23.4
16	Patient	7	Female	1.20	23	15.9
17	Healthy control	16	Female	1.65	63	23.0
18	Healthy control	17	Female	1.63	65	24.5
19	Healthy control	11	Male	1.60	48	18.7
20	Healthy control	14	Male	1.60	52	20.3
21	Healthy control	16	Female	1.70	63	21.8
22	Healthy control	17	Male	1.79	72	22.5
23	Healthy control	17	Female	1.70	70	24.2
24	Healthy control	15	Female	1.59	58	22.8
25	Healthy control	18	Male	1.69	85	29.8
26	Healthy control	18	Male	1.75	91	29.7
27	Healthy control	17	Male	1.73	70	23.4
28	Healthy control	18	Male	1.67	106	38.0
29	Healthy control	16	Female	1.71	59	20.2
30	Healthy control	14	Female	1.60	55	21.4
31	Healthy control	18	Female	1.52	57	24.7
32	Healthy control	17	Male	1.85	80	23.4
33	Healthy control	15	Female	1.79	97	30.3
34	Healthy control	18	Female	1.63	57	21.5
35	Healthy control	10	Female	1.45	42	20.0
