# Supplementary material for: Colon length in pediatric health and constipation measured using magnetic resonance imaging and three dimensional skeletonization
Source: PLoS One. 2024 Jan 2;19(1):e0296311. doi: 10.1371/journal.pone.0296311 (PMC10760671; doi:10.1371/journal.pone.0296311)
Supplement: Attachment — Submitted filename: Response to Reviewers.docx [file pone.0296311.s003.docx]

**PONE-D-23-27259 Revision R2**

**Colon length in pediatric health and constipation measured using magnetic resonance imaging and three dimensional skeletonization**

**Response to the Editor**

Thank you for your comments. We have addressed all the points raised as detailed below in this point-by-point response. All changes made to the manuscript are tracked in the tracked changes Word version enclosed.

We hope that the manuscript would now meet the journal’s criteria.

**Journal Requirements Comments:**

**Please review your reference list to ensure that it is complete and correct. If you have cited papers that have been retracted, please include the rationale for doing so in the manuscript text, or remove these references and replace them with relevant current references. Any changes to the reference list should be mentioned in the rebuttal letter that accompanies your revised manuscript. If you need to cite a retracted article, indicate the article’s retracted status in the References list and also include a citation and full reference for the retraction notice.**

Answer: We checked each reference individually as recommended and spotted an incorrect title and some typos in References 4, 10, 21 and 22 which are now corrected. Apologies for not seeing these before.

**Additional Editor Comments:**

**I believe that the authors have addressed the reviewers’ comments.**

Answer: Thank you.

**However, as can be seen from this revision, this study has several limitations. Considering these, it may be necessary to rephrase lines 302 to 305 in the conclusion.**

Answer: Agreed. We have rewritten that conclusion to stress that whilst providing new data that will help improve our understanding of colon pathophysiology and drug release modelling in children, the study also highlighted some fundamental limitations of the skeletonization technique that will need addressing to make the method more applicable in the future.

**Also, please consider whether it would be better to modify the content of the limitations and change where they are mentioned.**

Answer: We reconsidered the location of the limitations paragraph in the Discussion which appeared half-way through the more physio-pathological discussion. As such, we have now moved that section towards the end of the Discussion where such consideration are more traditionally placed. The content of the limitations was already quite comprehensive especially following the comments of the previous technical Reviewer. Accordingly we left the content unchanged.

**Figure Upload Comments:**

**While revising your submission, please upload your figure files to the Preflight Analysis and Conversion Engine (PACE) digital diagnostic tool,** [**https://pacev2.apexcovantage.com/**](https://pacev2.apexcovantage.com/)**. PACE helps ensure that figures meet PLOS requirements. To use PACE, you must first register as a user. Registration is free. Then, login and navigate to the UPLOAD tab, where you will find detailed instructions on how to use the tool. If you encounter any issues or have any questions when using PACE, please email PLOS at** [**figures@plos.org**](mailto:figures@plos.org)**. Please note that Supporting Information files do not need this step.**

Answer: We run again the three figure files through PACE, which found no image problems.
